# Eye Strain

**Published:** 1898-08

**Authors:** 


### EYE STRAIN.

Dr. Cheney has seen many cases with vertigo as a result of eye strain without other signs of asthenopia. It is frequent during middle life and is often regarded by the patient as a forerunner of apoplexy. Often the attack is of a very mild character, causing the patient but momentary discomfort; while in other cases it lasts for a number of minutes and is of sufficient severity to necessitate sitting or lying down. A mild feeling of dizziness, frequently accompanied by slight nausea, lasting for hours is another form of the trouble, but this is usually associated with prolonged use of the eyes, and its origin is correctly ascribed by the patient.

Drowsiness is not often recognized as a result of eye strain, but it is quite common, and is often unassociated with other eye symptoms. Several cases of this class are reported.

